# Intensified food production and correlated risks to human health in the Greater Mekong Subregion: a systematic review

**DOI:** 10.1186/s12940-015-0033-8

**Published:** 2015-05-26

**Authors:** Carsten H. Richter, Benjamin Custer, Jennifer A. Steele, Bruce A. Wilcox, Jianchu Xu

**Affiliations:** 1 Center for Mountain Ecosystem Studies, Kunming Institute of Botany, Chinese Academy of Sciences, Kunming, 650201 China; 2 Graduate University of Chinese Academy of Sciences, Beijing, 100049 China; 3 World Agroforestry Centre (ICRAF), East and Central Asia Region, Kunming, 650201 China; 4 Department of Infectious Disease and Global Health, Cummings School of Veterinary Medicine, Tufts University, North Grafton, MA 01536 USA; 5 Global Health Asia, Integrative Education and Research Programme, Faculty of Public Health, Mahidol University, Bangkok, 10400 Thailand

**Keywords:** Greater Mekong Subregion, Intensified food production, Agricultural intensification, Emerging diseases, Pesticides, Antibiotics, Livestock operations, Health promotion

## Abstract

**Background:**

Intensified food production, i.e. agricultural intensification and industrialized livestock operations may have adverse effects on human health and promote disease emergence via numerous mechanisms resulting in either direct impacts on humans or indirect impacts related to animal and environmental health. For example, while biodiversity is intentionally decreased in intensive food production systems, the consequential decrease in resilience in these systems may in turn bear increased health risks. However, quantifying these risks remains challenging, even if individual intensification measures are examined separately. Yet, this is an urgent task, especially in rapidly developing areas of the world with few regulations on intensification measures, such as in the Greater Mekong Subregion (GMS).

**Methods:**

We systematically searched the databases PubMed and Scopus for recent studies conducted on the association between agricultural (irrigation, fertilization, pesticide application) and livestock (feed additives, animal crowding) intensification measures and human health risks in the GMS. The search terms used were iteratively modified to maximize the number of retrieved studies with relevant quantitative data.

**Results:**

We found that alarmingly little research has been done in this regard, considering the level of environmental contamination with pesticides, livestock infection with antibiotic resistant pathogens and disease vector proliferation in irrigated agroecosystems reported in the retrieved studies. In addition, each of the studies identified focused on specific aspects of intensified food production and there have been no efforts to consolidate the health risks from the simultaneous exposures to the range of hazardous chemicals utilized.

**Conclusions:**

While some of the studies identified already reported environmental contamination bearing considerable health risks for local people, at the current state of research the actual consolidated risk from regional intensification measures cannot be estimated. Efforts in this area of research need to be rapidly and considerably scaled up, keeping pace with the current level of regional intensification and the speed of pesticide and drug distribution to facilitate the development of agriculture related policies for regional health promotion.

## Introduction

The Greater Mekong Subregion (GMS), founded by Cambodia, Laos, Myanmar, Thailand, Vietnam and Yunnan Province of China in 1992, is a bloc of rapidly developing and cooperatively linked economies that geographically share the Mekong River Basin [1]. The population, which includes various culturally and linguistically distinct ethnicities in addition to those primarily associated with each nationality exceeded 271 million in 2010 [2, 3]. China and Thailand are by far the most rapidly developing economies of the region with a steady increase in the demand for food quantity and diversity, but also for cash crops such as rubber. However, as arable lands are limited, the pressure on farmers and herders to scale up production grows in correlation with the socio-economic development.

**Fig. 1:**
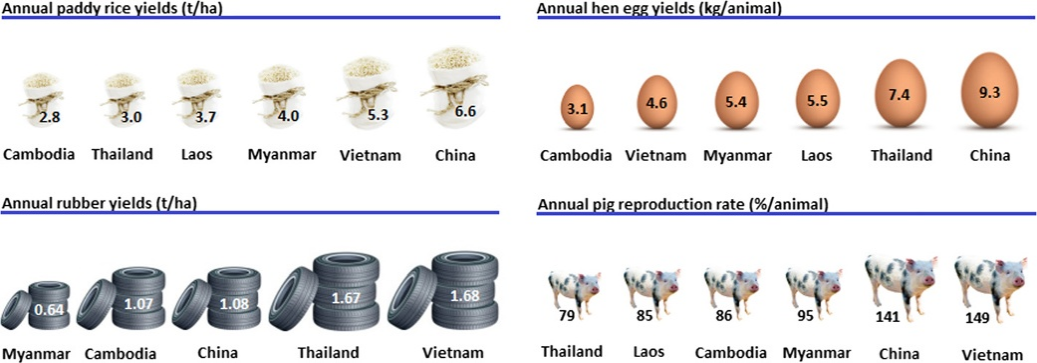
Productivity indicators as proximate measures for average national sector intensification, 5 year average 2007–2011 [2]. The pig reproduction rates consider annual increases in stock, slaughtered animals and import/export numbers

Consequently, farmers and herders facing land scarcity and a depletion of soil nutrients seek to intensify their operations [4]. Intensified food production aims for higher yields per area through an increase in inputs and operational efficiency. The main inputs constitute water, nutrients and pesticides in crop systems and feed supplemented with hormones and antibiotics in livestock operations. Efficiency is increased by specialization in context-dependent high-yielding species and mechanization [5]. Thereby, crop and livestock intensification exhibit different trends in areal expansion. While farming systems situated in the region’s core agricultural areas commonly are the first to intensify production [4], intensive livestock operations are not restricted to arable lands and thus concentrate around urban centers, minimizing the distance between production and markets [6, 7]. Figure 1 illustrates the variation in average productivity per area as a proximate of intensification among the 6 countries of the GMS.

Intensification measures in the region have been linked to ecological changes with adverse effects on human health [8]. For instance, irrigation schemes may expand the habitat of disease vectors thriving in and around water bodies [9], while the application of agrochemicals may result in a proliferation of populations of problematic primary consumers, such as mosquito larvae or vector snails in wetlands [10]. Large monocultures may serve as ample seasonal food sources to wildlife hosts of zoonotic pathogens such as rodents [11, 12]. This potentially promotes the emergence of zoonoses in three ways: directly through the proliferation of host populations, indirectly through the proliferation of vector populations feeding on these hosts, and by increasing the likelihood of encounters between wildlife and domestic animals or humans. In addition, human exposure to pesticides and fertilizer residues has been associated with the development of neurological disorders, immune suppression and reduced fertility [13–17].

Industrial livestock production systematically utilizes anabolic steroids and antibiotics as growth accelerators, potentially contaminating surface waters and meats [18–20] and promoting drug resistance in food-borne bacteria [21], a main contributor to the growing number of emerging infectious diseases [22]. Animal crowding supported by automated feeding systems increases the animal contact rate and induces immunosuppressive stress among them, both factors potentially promoting disease transmission. Concurrently, systematic breeding for uniformly high productivity decreases genetic variability and in turn increases individual susceptibility to infections and their likelihood of transmission within a herd [23, 24].

Despite these reported mechanisms and the general recognition of the links between intensified food production and disease emergence [25], quantifying the risks that individual measures or practices bear with respect to human health remains a challenge. Without the ability to quantify these risks, however, the obvious benefits of increased productivity cannot be appropriately balanced against the combined negative health implications of intensification measures. Thus, the objectives of this systematic review are to determine if individual measures of intensified food production in the GMS have been shown to cause quantifiable negative impacts on human health, to make known risks associated with current regional intensification more assessable, and to make the consequences of further intensification more predictable. The review will also identify critical research gaps concerning the relationship between intensified food production, human health risks and disease emergence. This is an urgent task at a time of rapid socio-economic development in a region that is largely lacking regulations and the capacity for controls of agricultural operations and quality and safety standards for agricultural products.

## Methods

References for this review were identified through searches of the databases PubMed and Scopus in July-August 2014. Search results containing original research with quantitative data on intensified food production and/or its effects on human health in the GMS were considered relevant. No result was excluded for its language. For reasons of contemporary validity, only studies conducted since the year 2000 were included.

To determine a set of appropriate search terms we began with the most obvious combinations of “intensified food production”, “agricultural intensification”, “livestock production”, “human health” and “Greater Mekong Subregion”. However, the combination of these terms was without result. Therefore, we detailed the regional scope by the Boolean statement: “Cambodia” OR “Laos” OR “Myanmar” OR “Thailand” OR “Vietnam” OR “Yunnan”. This specification resulted in a small number of irrelevant papers, either of purely conceptual nature or reporting data from before the year 2000. Thus, we went forward by detailing the predominant types of inputs for crops: “irrigation”, “fertilizers” and “pesticides”, and for livestock: “antibiotics” OR “antimicrobials, “hormones” and “feeding technology”. This yielded some results for the crops aspects, however because the results for “irrigation” and “human health” did not contain studies on vector proliferation, we added another search term combination: “vectors” and “irrigation”.

Because the search terms for the livestock aspects resulted in very few articles, we replaced the rather general “human health” term with the products of livestock operations, namely “meats” OR “eggs” OR “dairy” to investigate on human health impacts from feed supplements throughout the food value chain. This resulted in considerably more results and relevant studies, though in respect to antibiotics in meats only. Other concerns of contamination with residues from supplemented feeds, such as fecal discharge in waters or as fertilizers on fields thereby were expected to be reflected by the crop search terms. As for search terms such as “growth promotants” or “hormones” this combination of terms still did not yield any relevant results. However, antibiotics are also used as growth promotants in livestock operations, such that this aspect is partially included in those results.

Combinations with the search term “feeding technology” did not yield any further results, but feeding technology is relevant as it makes grazing unnecessary and enables animal crowding. Resulting close and frequent contacts among animals potentially promote the spread of infectious disease, including zoonoses. Thus, we replaced the terms for livestock products with “zoonoses”. Because this still did not yield any results, we also replaced the rather specific term of “feeding technology” with the more general term “animal production” with more success (the terms “livestock density” and “animal density” did not yield any results).

The final choice of search terms and their combinations are shown in Table 1, with three different search terms for inputs to agricultural and livestock systems each and a search term addressing the respective human health aspect, from the general term “human health”, to livestock products focusing on risks for the end consumers and the risk of the spread of “zoonoses” due to operational efficiencies (the term “zoonosis” yielded the same results, the term “zoonotic” yielded no results). Table 1 lists the number of respective search results, excluded and included studies from the GMS, as well as search results globally.

**Table 1:** Summary of systematic review methodology and results

Search terms per review focus		Mekong region						Global scope		
Health aspect	Intensification aspect	No. of results PubMed Scopus		No. of unique results	Exclusion criteria			No. of relevant studies	No. of results PubMed Scopus	
					Different study focus	No relevant quantitative data	Study from before 2000, or year not indicated			
“vectors”	“irrigation”	2	8	8	2	–	4	2	271	658
“human health”	“irrigation”	2	8	9	4	3	1	1	137	462
	“fertilizers”	1	5	5	3	2	–	0	99	552
	“pesticides”	15	31	36	13	8	6	9	1,014	1,957
“meats” OR “eggs” OR “dairy”	“antibiotics” OR “antimicrobials”	6	65	66	21	16	10	19	1,241	9,564
	“hormones”	9	7	14	11	–	3	0	4,352	10,214
“zoonoses”	“animal production”	3	3	4	1	1	–	2	72	96

The table includes search terms and the number of results per database, the number of unique results, of excluded article per criteria and the number of relevant studies for this review. The table also lists the number of search results considering a global scope for comparison

## Results

### Intensification in crop systems

#### Irrigation

In spite of the obvious linkages between irrigation structures and the proliferation of disease vectors such as mosquitoes inhabiting irrigated areas, this review identified only two studies on freshwater snails of medical importance. Sri-Aroon et al. [26] conducted a snail sampling study at 6 sites within the irrigation system of Lam Pao Dam in Kalasin Province, Thailand in December 2003. They found a total of 15 snail species, including 7 species with suspected involvement in human infections of angiostrongyliasis, cercarial dermatitis, echinostomiasis, opisthorchiasis and paragonimiasis. Snails of the species *Bithynia siamensis goniomphalos*, which are potential intermediate hosts of the liver fluke *Opisthorchis viverrini* accounted for 89 % of all collected samples, and exhibited a high infection rate compared to studies from endemic areas in Khon Kaen province, Thailand.

Meanwhile, an interventional study in Yunnan province, China found that the implementation of a water-saving irrigation project reduced the abundance of snails of the genus *Oncomelania* to about 5 % [27]. Snails of this genus are potential vectors of flukes causing schistosomiasis in humans. The project was followed by a decline in human schistosomiasis infections in that area.

In addition, one study reported quantitative data and gave an estimate for the risk of diarrheal disease, associated with the consumption of raw vegetables (100 g per person per week) contaminated with enteric pathogens from irrigation water. This study was conducted in a peri-urban area of Thailand where farmers draw water for irrigation from a crosshatched network of canals that are also used for wastewater disposal. Using Monte Carlo simulations, Diallo et al. [28] estimated the combined risk of diarrheal disease in that area due to dietary contamination by the protozoan parasites *Cryptosporidium hominis*, and *Giardia lamblia*, and the bacteria *Escherichia coli* at between 0.56 and 0.62 per person per year.

#### Fertilizers

Fertilization is a critical component for increasing yields. However, its uncontrolled application can result in a loss of biodiversity [29] and negative effects on human health. High nitrate concentrations in drinking water from over-fertilization have been associated with methemoglobinemia and consequential cyanosis in infants [15]. Nitrate concentration from fertilizer runoff was estimated at 3.5 g/l at the mouth of the Mekong and at 5.5 g/l at the mouth of the Chao Phraya rivers in 2000 [30]. However, within the scope of this review no study was identified quantifying nitrate concentrations in local water sources and relative risk to human health.

In addition, potentially pathogenic livestock manure is frequently used as fertilizer in integrated farming systems [31], creating a zoonotic risk associated with contaminated produce. In Vietnam, an experimental study investigated the local spread of antibiotic resistance from pig manure to organisms of the water environment in integrated pig-fish farms [32]. However, this review did not identify any study quantifying the relationship between particular fertilization practices and resulting disease emergence.

#### Pesticides

In agriculture, pesticides are substances that are applied to crops to protect them from organisms that have the potential to reduce yields. Pesticides, which can be of biological or synthetic origin, become an issue to human health when their yield-protecting toxicity exhibits harmful effects beyond their target, either directly through human exposure or indirectly through ecosystem degradation. During the 10 years from 1997–2007, almost 1100 new types of pesticides (differing in active ingredients or formulation) were registered in Vietnam [33] while pesticide use by weight grew at an annual average of 7.6 % between 1990 and 2003 [34].

Within the scope of this review however, only one comprehensive study on the range of applied compounds was identified, raising particular concern about residues of carbamates, organochlorines, organophosphates, and pyrethroids in foods and the environment [35]. This study lists 40 different products containing at least 36 different compounds that were being sold to farmers in northern Vietnam from 2006–2009. The agricultural utilization of 2 of these compounds (endosulfan and DDT) has been banned by the member states of the Stockholm Convention, a global treaty aiming for the protection of human health from Persistent Organic Pollutants (POPs). All of the countries of the GMS had ratified the Stockholm Convention by the end of 2006. However, due to weak government enforcement, retailers and farmers have been reported to frequently break pesticide regulations [33].

As pesticide users, farmers constitute a primary risk group with respect to pesticide exposure. Timprasert et al. [36], conducting a survey with vegetable farmers in Nakhon Ratchasima Province of Thailand in 2008/2009 found that most farmers applied synthetic pesticides every week as a protective measure. Two of the reasons given by farmers for using synthetics were convenience of application and low labor requirements. However, farmers are often unaware of health and environmental impacts from improper application, storage and disposal of hazardous pesticides and unable to protect themselves and their families from direct or indirect contact [37, 38]. Phung et al. [39] measured farmers’ exposure to the insecticide chlorpyrifos, which can cause cholinesterase inhibition, by analyzing urinary samples from a group of rice farmers in Vietnam. The detected levels of pesticide uptake in this group exceeded international safety levels and likely caused adverse health effects. Furthermore, a survey among fishermen in Pathum Thani, Thailand found that dermal exposure to pesticide residues in surface waters constitutes an additional risk factor for carcinogenesis among this occupational group [40].

Apart from occupational exposure, consumers of regional drinking water, produce and aquatic organisms constitute the largest group potentially at risk of pesticide contamination. In spite of the diversity and potential hazardousness of chemical compounds being applied to crops and soil, only 3 studies on residue contamination of drinking water or foods in the region were identified (Table 2). A study on the contamination of rivers and lakes with banned organochlorine pesticides (OCPs) in Yunnan in 2003 [41] detected great differences among tested inland waters, ranging from 0.38 μg/l in Lake Chenhai up to 24.53 μg/l in Lake Dianchi. Furthermore, the authors state that an increasing number of people living in cities and towns along those waterbodies are using them directly as sources of drinking water. Accordingly, regional populations relying on surface water as a source of drinking water may already be at considerable risk of developing symptoms associated with the long-term consumption of local pesticide residues.

**Table 2:** Studies on pesticide residues in drinking water and aquatic organisms oral Reference Doses (RfD)

Study scope			Sampling results		Daily intake of pesticide residues (% of RfD^a^)											
Contamination	Geography (province, country)	Period	Biomass conside-rations	Data repre-sentation	DDT, DDD, DDE	HCB	α-HCH	β-HCH	γ-HCH	Endo-sulfan	Hepta-chlor	Cyflu-thrin	Cyper-methrin	Feno-bucarb	Trichlor-fon	Dieldrin
Inland waters [41]	Yunnan, China	2003	Adult^b^	Range	0-13 %	0-13 %	0-12 %	0-65 %	0-409 %							
				Average	1.5 %	2.5 %	2 %	21 %	75.5 %							
			Child^c^	Range	0-37 %	0-37 %	0-34 %	0-188 %	0-1180 %							
				Average	4.5 %	6.5 %	5.5 %	61.5 %	218 %							
Fresh water organisms (vegetables, invertebrates, fish) [42]	Pathum Thani, Thailand	2004-2007	Ages 10–75 (mean 36)	Range^d^	13.5-31 %			4-9.5 %		1-2.5 %	4.5-10 %					
				Average^e^	22.5 %			7 %		2 %	7.5 %					
Pond fish [43]	Hanoi, Vietnam	2007-2008	Adult	Range^f^	0.5 %							0-0.5 %	0.5-3 %	0 %	0-0.5 %	4.5-40.5 %
				Average^f^	0.5 %							0 %	1.5 %	0 %	0 %	15 %
	Phu Tho, Vietnam			Range^f^	0.5-4 %							0-0.5 %	0.5-3.5 %	0 %	0-5.5 %	4.5-12.5 %
				Average^f^	1.5 %							0.5 %	1.5 %	0 %	2 %	7.5 %

^a^The Reference Dose (RfD) is an estimate of a daily human exposure (including sensitive subgroups) that is likely to be safe over extended periods of time; numbers are rounded to the nearest 0.5 %

^b^Average adult weight (Asia) of 57.7 kg [96] and 2 l of daily drinking water consumption [97]

^c^Daily drinking water consumption of 1 l at a children weight of 10 kg [97]

^d^Considers the range in local consumption of 2 standard deviations around the mean [42]

^e^Average weight of study participants of 59 kg [42]

^f^Based on an average Vietnamese diet (Hanoi) [84]

In addition, two studies from Thailand [42] and Vietnam [43] measured the contamination of aquatic organisms with pesticide residues and detected concentrations at levels that are potentially harmful at daily consumption rates of local diets. To objectively evaluate the risks stated in these 3 studies, Table 2 translates reported contaminations to percentages of the oral Reference Dose (RfD) per respective compound based on local diets. Table 3 lists the potential hazards associated with an exposure exceeding RfDs of those compounds included in the three studies and their risk classification according to the World Health Organization [44].

**Table 3:** Overview of toxic hazards and respective oral RfDs of pesticide residues quantified by identified studies

Compound	Metabolites	Agricultural use	Human hazard	Human oral RfD	GHS class^a^
Carbamates					
Fenobucarb		Insecticide	Neurotoxic [98]	60 μg/kg/day [99]	4
Organochlorines					
Aldrin		Insecticide	Hepatotoxic [100]	0.03 μg/kg/day [100]	–
	Dieldrin		Probably carcinogenic [100]	0.05 μg/kg/day [100]	–
Dichlorodiphenyl-trichloroethane (DDT)	Dichlorodiphenyl-dichloroethane (DDD), Dichlorodiphenyl-dichloroethylene (DDE)	Insecticide	Neurotoxic [101]	0.5 μg/kg/day [100]	3
			Hepatotoxic [100]		
			Probably carcinogenic [100]		
			Endocrine-disrupting [101]		
Endosulfan		Insecticide	Neurotoxic [102]	5 μg/kg/day [102]	3
			Nephrotoxic [102]		
Heptachlor		Insecticide	Probably carcinogenic [100]	0.5 μg/kg/day [100]	–
	Heptachlor epoxide			0.013 μg/kg/day [100]	–
Hexachlorobenzene (HCB)		Fungicide	Neurotoxic [103]	0.8 μg/kg/day [100]	5
			Hepatotoxic [100]		
			Probably carcinogenic [100]		
α-Hexachloro-cyclohexane (α-HCH)		Insecticide	Neurotoxic [103]	8 μg/kg/day [104]	3
			Hepatotoxic [100]		
			Probably carcinogenic [100]		
β-Hexachloro-cyclohexane (β-HCH)		Insecticide	Neurotoxic [103]	0.6 μg/kg/day [104]	3
			Possibly carcinogenic [100]		
γ-Hexachloro-cyclohexane (γ-HCH)		Insecticide	Neurotoxic [103]	0.01 μg/kg/day [104]	3
			Hepatotoxic [100]		
			Nephrotoxic [100]		
			Potentially carcinogenic [103]		
Organophosphates					
Trichlorfon		Insecticide	Neurotoxic [105]	2 μg/kg/day [105]	3
Pyrethroides					
Cyfluthrin		Insecticide	Nephrotoxic [100]	25 μg/kg/day [100]	2
Cypermethrin		Insecticide	Neurotoxic [100]	10 μg/kg/day [100]	3

^a^UNECE’s “The Globally Harmonized System of Classification and Labeling of Chemicals” (GHS): 1, 2 - Fatal if swallowed; Fatal in contact with skin, 3 - Toxic if swallowed; Toxic in contact with skin, 4 - Harmful if swallowed; Harmful in contact with skin, 5 - May be harmful if swallowed; May be harmful in contact with skin [44]

### Intensification in livestock systems

#### Antibiotic resistance

While the use of antibiotics for humans is unrestricted in Thailand, the use for animals has been regulated by the Thai Department of Livestock Development since 2003, with varying degree of compliance among farmers [45]. A study conducted in Sangkhla Buri, Kanchanaaburi provice, Thailand in 2002/2003 found enteric bacterial pathogens in 97 % of raw food samples (chicken, pork and fish) from a local market. The two most common bacteria identified in this study were *Salmonella* (84 %) and *Arcobacter butzleri* (74 %), followed by *Campylobacter* (51 %), *Plesiomonas* (27 %) and *Aeromonas* (5 %) [46]. Similarily, Van et al. [47] probed Vietnamese retail meat including pork, beef and chicken and found *Salmonella* in more than 60 % and *Escherichia coli* in more than 90 % of all samples.

Table 4 lists studies on antibiotic resistance of *Salmonella* from chickens, pigs and cattle (including the top 10 antibiotics with reported rates of resistance), conducted in the GMS since 2000 (studies were included if they were animal species specific and indicated the sampling year). A study from Khon Kaen province, Thailand, 2003, found the rates of antibiotic specific resistance of *Salmonella* in pork, chicken, and human patients to be closely related [48], suggesting a transfer of resistant bacteria through the food chain to the consumer. It has been estimated that there were about 22.8 million cases of gastroenteritis caused by *Salmonella* alone in Southeast Asia in 2006, with about 37,600 fatalities [49].

**Table 4:** Antibiotic resistance of *Salmonella* in animals and meats in the GMS

Year	Sample origin	Province, country	Sample size	Percentage of resistance										Source
				TET	AMP	NAL	CEP	STR	CHL	SXT	FLO	SUL	AMX	
Chicken														
2012	Wet market and super-market	Ha Noi, Ho Chi Minh, Phu Tho and Lam Dong, Vietnam	457	59	42	–	–	–	37	35	–	–	–	Ta et al., 2014 [50]
2010-2011	Super-market	Bangkok, Thailand	14	21	79	–	0	–	–	17	–	–	–	Chaisatit et al., 2012 [57]
2010	Retail market	Phatthalung, Thailand	38	60	68	76	5	92	68	5	–	–	–	Lertworapreecha et al., 2013 [106]
2004	Market and supermarket	Ho Chi Minh City, Vietnam	18	39	22	39	0	28	11	–	–	–	22	Van et al., 2007 [47]
2003	Retail market	Khon Kaen, Thailand	30	100	–	–	–	100	27	20	–	100	30	Angkititrakul et al., 2005 [48]
2000-2002	Farm (fecal)	Chiang Mai and Lamphung, Thailand	11	100	0	100	–	–	–	–	27	–	–	Padungtod and Kaneene, 2006 [51]
	Market		57	33	0	43	–	–	–	–	0	–	–	
	Slaughter-house		87	16	0	16	–	–	–	–	0	–	–	
2000-2001	Wet market	Mekong Delta, Vietnam	20	–	5	35	–	20	25	–	–	–	–	Ogasawara et al., 2008 [107]
Pigs and Pork														
2011	Market	Champasak, Laos	35	63	60	14	–	57	11	37	–	–	–	Boonmar et al., 2013 [108]
2010	Retail market	Phatthalung, Thailand	45	77	51	4	28	71	11	17	–	–	–	Lertworapreecha et al., 2013 [106]
2010	Farm (fecal)	Sa Kaew, Thailand	3	33	33	0	–	33	33	0	–	–	–	Pulsrikarn et al., 2013 [109]
	Retail market		42	69	50	0	–	31	14	36	–	–	–	
2004	Market and supermarket	Ho Chi Minh City, Vietnam	32	78	50	25	0	16	0	–	–	–	50	Van et al., 2007 [47]
2003	Retail market	Khon Kaen, Thailand	26	89	–	–	–	100	15	15	–	100	15	Angkititrakul et al., 2005 [48]
2000-2001	Farm (fecal)	Chiang Mai and Lamphung, Thailand	51	98	0	2	–	–	–	–	6	–	–	Padungtod and Kaneene, 2006 [51]
	Market		155	60	0	21	–	–	–	–	8	–	–	
	Slaughter-house		48	89	1	39	–	–	–	–	15	–	–	
2000-2001	Wet market	Mekong Delta, Vietnam	48	–	6	–	–	15	13	–	–	–	–	Ogasawara et al., 2008 [107]
Cattle and Beef														
2011	Market	Champasak, Laos	20	75	70	5	–	80	15	30	–	–	–	Boonmar et al., 2013 [108]
2009	Retail market	Hanoi, Vietnam	63	46	32	18	–	30	22	–	–	–	–	Thai et al., 2012 [110]
2005-2007	Farm (fecal)	Nakhonpathom, Thailand	160	9	4	–	–	64	2	–	–	11	–	Chuanchuen et al., 2010 [111]
2004	Market and supermarket	Ho Chi Minh City, Vietnam	32	13	0	6	0	6	0	–	–	–	0	Van et al., 2007 [47]
2000-2001	Wet market	Mekong Delta, Vietnam	35	–	–	–	–	6	3	–	–	–	–	Ogasawara et al., 2008 [107]

*TET* tetracycline, *AMP* ampicillin, *NAL* nalidixic acid, *CEP* cephalothin, *STR* streptomycin, *CHL* chloramphenicol, *SXT* trimethoprim/sulfamethoxazole, *FLO* florfenicol, *SUL* sulfamethoxazole, *AMX* amoxicillin

In a recent study from Vietnam, Ta et al. [50] differentiated between chicken from small free-range backyard production without medicated feed and large enclosed operations with feed possibly containing antibiotics. However, the authors found no significant difference in antibiotic resistance of *Salmonella* between these two livestock production systems.

In addition, Padungtod et al. [51] tested samples (fecal and meat) from chickens, pigs and dairy cattle for presence and antibiotic resistance of *Campylobacter* bacteria in Chiang Mai and Lamphung provinces, Thailand, from 2000–2002. *Campylobacter* isolates were present in approximately 60 % of the samples and the authors detected high resistance to ciprofloxacin (65 %), nalidixic acid (74 %) and tetracycline (66 %) as average over all livestock samples, as well as to erythromycin (71 %) and azithromycin (70 %) as average over all pig samples. Similarily, Noppon et al. [52] obtained 294 *Campylobacter* isolates from chicken meats in Khon Kaen province, Thailand in 2007/2008 and detected *C. jejuni* resistance to ofloxacin (91 %), doxycycline (37 %), erythromycin (29 %) and chloramphenicol (13 %). Regarding humans, children below the age of 2 years are at highest risk of developing gastroenteritis following *Campylobacter* infection [53]. Isolation rates from diarrheal children below the age of 5 years were previously estimated at 13 % in Thailand [54] and 12 % in Laos [55].

Furthermore, in a study on antibiotic resistance of *Escherichia coli* bacteria in food samples (including beef, chicken, pork and shellfish) from markets and supermarkets around Ho Chi Minh City, Vietnam in 2004, Van et al. [56] detected resistance to tetracycline (78 %), sulfafurazole (61 %), ampicillin (51 %), amoxicillin (51 %), trimethoprim (52 %), chloramphenicol (43 %), streptomycin (39 %), nalidixic acid (34 %), gentamicin (24 %), ciprofloxacin (16 %), norfloxacin (17 %) and enrofloxacin (21 %) in tested isolates. In a similar study on chicken meat samples from supermarkets in Bangkok, Thailand in 2010/2011, Chaisatit et al. [57] detected resistance to ampicillin (72 %), tetracycline (49 %), gentamicin (38 %), cephalothin (28 %), chloramphenicol (17 %), ciprofloxacin (11 %), levofloxacin (11 %), trimethoprim-sulfamethoxazole (7 %), amoxicillin-clavulanic acid (6 %) and cefuroxime (2 %). Verotoxins from *Escherichia coli* can cause diarrhoea, haemorrhagic colitis and haemorrhagic uraemic syndrome [58].

Finally, a study conducted on meats from supermarkets and open markets in Bangkok in 2007 found *Listeria monocytogenes* in 15.4 % of all samples (n = 104). Of these isolates, 95.5 % were resistant to cefotaxime, ceftazidime and ceftriaxone [59]. Serious manifestations of human listeriosis infection include septicaemia, encephalitis, meningitis, spontaneous abortion and stillbirth [60–63].

#### Hormones

Despite the large number of global studies conducted on hormonal residues and their possible impact on human health (Table 1) this review did not identify any relevant study from the GMS.

#### Livestock industrialization and zoonosis

In commercial livestock operations animals are kept in large groups at high density, which potentially facilitates intra- and interspecies transmission of pathogens. Farm workers, veterinarians and their families constitute a high risk group for zoonotic infections in such specialized facilities due to the high animal-human contact rate and the variety in exposure (inhalation, ingestion, dermal contact) implied in these operations. In comparison to small-scale operations such as from backyard producers, commercial livestock production is commonly associated with higher biosecurity. However, Graham et al. [64] tested this hypothesis on data of the highly pathogenic avian influenza (HPAI) outbreaks in Thailand in 2004 and found no supporting evidence. While the greatest number of outbreaks in 2004 was reported from backyard flocks, this figure does not account for relative risk, considering that the majority of flocks are still backyard operations with a vastly smaller number of potentially infected animals per flock.

In addition to the risks associated with crowding, animals of commercial operations may constitute a higher risk for human infection when their relatively young age at time of slaughter does not suffice for developing immunity to common farm pathogens. For example, *Campylobacter* infections are an emerging zoonosis [22] of this kind. In commercial broiler farms in Vietnam, animals are typically slaughtered after 5–6 weeks, i.e. at half the age compared to animals from backyard and semi-intensive operations, just at the peak of *Campylobacter* infection prevalence [65–67]. Accordingly, Carrique-Mas et al. [68] investigated the risk of *Campylobacter* infections of pigs and poultry held in operations of small to large size, in the Mekong delta region of Vietnam and identified animal age as a primary risk factor for *Campylobacter* infection in chickens and pigs, with a high rate of immunity in older animals.

## Discussion

This systematic literature review revealed substantial knowledge gaps regarding the quantitative effects of intensified food production on human health and disease emergence in the GMS. We found that in spite of the now widely recognized links between environmental change from agricultural intensification and disease emergence [69–72], research aiming at quantifying relationships between agricultural inputs and potential adverse effects to human health exhibits an almost exclusive focus on pesticides (environmental and medical). Only few studies look for indirect implications of development on the abundance of disease vectors, the epizootic potential of livestock and the potential of zoonotic disease emergence. Yet, evidence of the correlation between agricultural intensification and biodiversity loss and possible influences on disease emergence in this region is growing [73, 74]. This includes studies on the composition of soil microorganisms after repeated use of the herbicide glyphosate, promoting the relative abundance of gram-negative bacteria, such as *Burkholderia* spp., which have been linked to the regional emergence of human melioidosis [75, 76].

The majority of studies included in this review address the issue of emerging antibiotic resistance in regional bacteria strains, but only a few studies investigate the relationships between the number and density of animals and disease emergence in different types of production systems. While larger operations are commonly associated with a higher level in biosecurity, this notion may largely be influenced by a statistical bias in data representation [64]. On the other hand, Ta et al. [50] did not find a significant difference in antibiotic resistance among chickens from free-range backyard operations and enclosed units utilizing antibiotic feed. Similarly, regional studies on antibiotic resistance of various bacteria in meats could not establish a correlation between the origin of the samples and their level of resistance exhibited (Table 4). Further interdisciplinary research is needed to investigate these relationships, which may be much more complex than commonly thought.

However, what becomes obvious from these studies nonetheless, is that antibiotic resistant bacteria are a widespread phenomenon in livestock and animal products throughout the GMS. Common human enteric pathogens such as *Salmonella, E. coli and Campylobacter* are found in both live animal samples and meats for purchase, and these strains exhibit extensive antibiotic resistance, often to multiple drugs. Antibiotic resistance results from the use of antibiotics as growth promotants in animal feeds and inappropriate use of antibiotics for treatment of livestock in systems of any scale. In addition, this review highlights the contribution of livestock intensification to the transmission of resistant strains through the food chain. Foodborne organisms already contribute significantly to human morbidity and mortality and per capita meat consumption will further increase in correlation with regional development.

These issues associated with intensifying livestock operations have been recognized and investigated in developed regions including the United States and European Union for decades [77]. DuPont et al. called for surveillance of antibiotic resistance in 1987 [78], and numerous studies have since recommended to prohibit the practice of misusing antibiotics for non-therapeutic purposes [70, 79–82]. Consequentially, the European Union banned the use of antibiotics in livestock feed in 2006, while the United States Food and Drug Administration has issued voluntary guidance to reduce the use of antibiotics in livestock feed, a notion supported by the World Health Organization (WHO), the Food and Agricultural Organization of the United Nations (FAO), and the World Organization for Animal Health (OIE) [83]. In addition to new insights from upscaled disciplinary and interdisciplinary research efforts, these international guidelines could provide a basis for national or regional policy reforms in the GMS.

This review identified only a few studies investigating human dietary pesticide exposure through contaminated food and drinking water in the GMS (Table 2). Collectively, these studies merely account for a small percentage of the 406 hazardous active pesticide ingredients listed by the WHO [44] and for a small percentage of average dietary intake by food diversity and weight [42, 84]. However, even this limited research suggests potentially hazardous contamination of food and drinking water in the region, especially if considering the possibility of additive and synergistic effects.

In addition to continuous dietary exposure to pesticide residues, the Poison Control Center in Hanoi reported 168 admitted cases of direct pesticide poisoning from 1999 to 2003 [85]. However, few poisoning incidents caused by pesticides are detected by the health care system. A self-surveillance study of 50 farmers in Vietnam found an underreporting rate of 96 % corresponding to untreated symptoms [86], while the WHO reported 7,170 cases of acute pesticide poisoning in Vietnam in 2002 [87]. In combination, these figures could thus accumulate to almost 180,000 cases of pesticide poisoning of various degree per year in Vietnam alone. Furthermore, due to the growing market diversification brought about by the great number of annual registration of new products and compounds, risks associated with direct or indirect pesticide exposure are naturally becoming more complex and are continuously shifting. For instance, in 2004 a high incidence of poisoning from exposure to organophosphates and carbamates was reported by the World Bank [88]. In 2012, the majority of fatalities directly linked to pesticides at the Poison Control Center in Hanoi were reported as paraquat poisoning [89].

In spite of these findings, direct and indirect impacts of environmental pesticides on human health remain vastly under-researched and under-reported. This can be observed in general, as risk evaluations and acceptable exposure doses are consistently only described for individual compounds (Table 3) without considering additive or synergistic effects among multiple compounds [16, 90]. To cope with the overwhelming number of possible compound combinations, Squillace et al. [91] suggested the conduct of toxicology studies on common mixtures of contaminants in water, an approach that could also be applied to various food categories. In addition, pesticide toxicity should also be evaluated in integrated studies that include synergistic effects among common risk factors beyond pesticide exposure [92].

However, proper problem specific integration of research within systemic, transdisciplinary programs remains a challenge of its own [93]. There is a ubiquitous discrepancy between conducting meaningful research and a limited availability of therefore required resources and capabilities. To accommodate this challenge, standardizations in operational criteria and research protocols for ecosystem approaches have recently been proposed, with the aim to increase comprehensiveness of research and intervention efforts across multiple or subsequent projects [94]. However their value significantly depends on the ability and disposition of individual researchers and institutions to collaborate on problems across disciplinary, geographic and institutional boundaries, as well as over time. In this context, the new regional development aims of communicable disease control as well as food and drug safety included in the GMS Strategic Framework and Action Plan 2012–2022 [95] may constitute a unique opportunity to promote new models of and incentives for scientific collaboration on these issues throughout the region.

## Conclusions

This review found significant evidence of substantial environmental contamination with pesticides with potentially serious health implications for local populations. In addition, bacterial contamination of meats and produce exhibiting high levels of resistance to a range of antibiotics is a widespread phenomenon in the GMS. Consequently, regional intensified food production has a broad potential impact on human health, ranging from acute to chronic poisonings and from occupational to food-borne infections.

Furthermore, we found that the number of identified studies by far does not match the seriousness of the risks to regional health. Because the specific types and amounts of agricultural inputs are locally variable with potential impact on distinct people, the few studies identified in this review hardly qualify for regional generalization but rather for raising additional attention to an alarming development. More disciplinary as well as interdisciplinary research is urgently needed to investigate and quantify local relationships between agricultural inputs and the emergence of zoonotic, food-borne and vector-borne diseases, as well as the risks of physical and mental impairment from chronic exposure to agricultural chemicals. This research should be planned and conducted in a systematic way to generate complementary data that allows an integrated evaluation of the regional level of intensification and correlated risks to human health over time, and thus a comprehensive tangible basis for the development of respective health promoting policies.
